# CYP17 blockade by abiraterone: further evidence for frequent continued hormone-dependence in castration-resistant prostate cancer

**DOI:** 10.1038/sj.bjc.6604904

**Published:** 2009-02-17

**Authors:** J E Ang, D Olmos, J S de Bono

**Affiliations:** 1 The Royal Marsden NHS Foundation Trust, Downs Road, Sutton, Surrey SM2 5PT, UK; 2 The Institute of Cancer Research, 15 Cotswold Road, Sutton, Surrey SM2 5NG, UK

**Keywords:** abiraterone, castration-refractory prostate cancer, androgen synthesis, CYP450c17, 17*α*-hydroxylase/C17,20-lyase

## Abstract

The limited prognosis of patients with castration-resistant prostate cancer (CRPC) on existing hormonal manipulation therapies calls out for the urgent need for new management strategies. The novel, orally available, small-molecule compound, abiraterone acetate, is undergoing evaluation in early clinical trials and emerging data have shown that the selective, irreversible and continuous inhibition of CYP17 is safe with durable responses in CRPC. Importantly, these efficacy data along with strong preclinical evidence indicate that a significant proportion of CRPC remains dependant on ligand-activated androgen receptor (AR) signalling. Coupled with the use of innovative biological molecular techniques, including the characterisation of circulating tumour cells and *ETS* gene fusion analyses, we have gained insights into the molecular basis of CRPC. We envision that a better understanding of the mechanisms underlying resistance to abiraterone acetate, as well as the development of validated predictive and intermediate endpoint biomarkers to aid both patient selection and monitor response to treatment, will improve the outcome of CRPC patients.

## AR-signalling pathway as a therapeutic target

Although the majority of patients diagnosed with prostate cancer are cured with definitive primary treatment, prostate cancer remains the second leading cause of male cancer-related death in the western world (Ferlay *et al*, 2007). Despite major advances made in the management of advanced prostate cancer, attributable mainly to the introduction of androgen-deprivation therapy (ADT) with most remissions lasting 2–3 years at best, many patients will eventually succumb due to the emergence of castration resistance (Pienta and Bradley, 2006). The centrality of androgens and the androgen receptor (AR) in castration-resistant prostate cancer (CRPC) is, paradoxically, echoed by the common, albeit erroneous, use of the terms ‘hormone resistant’ and ‘androgen independent’. Indeed, the progression of prostate cancer despite the administration of ADT is more a reflection of the ineffectiveness of current treatments than the disease gaining genuine autonomy from AR-signalling pathways.

The recognition that the state of androgen resistance is relative is certainly not new. Clinical experience in the management of breast and prostate cancers have clearly illustrated that repeated hormonal manipulations can be used successfully (Raghavan and Klein, 2008). Moreover, the importance of non-gonadal sources of androgens (adrenal and intracrine *de novo* synthesis) has long been recognised. In fact, contemporaneous ADT does not eliminate androgen synthesis within prostate cancer cells. On the contrary, intraprostatic (*in situ* and metastatic) levels of DHT and testosterone have been shown to remain elevated despite castrate serum levels (Titus *et al*, 2005). Additionally, not only is there autonomous overexpression of enzymes key to the synthesis of androgenic steroids (Stanbrough *et al*, 2006), AR-signalling pathways have also been shown to be persistently activated (Titus *et al*, 2005). All these point to the *de novo* intratumoural synthesis of androgens that could be critical to driving the progression of castration-resistant tumours. This may be compounded by the emergence of a hypersensitive phenotype (likely though AR mutation, amplification, and/or AR modulation by signalling pathways) that renders these cells exquisitely sensitive to extremely low levels of exogenous androgens (Taplin, 2007). Furthermore, currently approved antiandrogens, such as bicalutamide, hydroxyflutamide and nilutamide, have weak agonistic effects in prostate cancers with mutated or overexpressed AR (Chen *et al*, 2004), and this provides a mechanistic explanation for the treatment failure using these agents as well as the clinical phenomenon of the ‘androgen withdrawal response’. Overall, these data supported the study of a systemic CYP17 inhibitor to rationally deplete intratumoural and other extragonadal sources of steroid ligands to AR and ER*α* (androgenic and oestrogenic) that may impact CRPC biology.

## Inhibition of CYP17 using abiraterone acetate

### CYP17 in androgenic steroidogenesis

**Figure 1:**
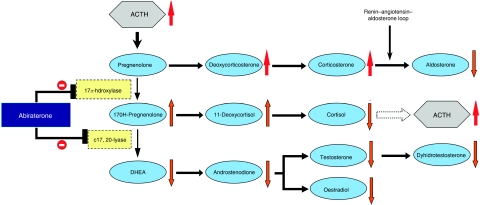
Androgen biosynthesis pathway. The physiological effects of abiraterone acetate on steroidogenesis are indicated by arrows next to each steroid precursor. Abiraterone acetate inhibits 17*α*-hydroxylase (blunt arrow), causing a decline in serum cortisol and a consequent rise in adrenocorticotrophic hormone (ACTH) (broken arrow). This, in turn, results in the rise of deoxycorticosterone and corticosterone by approximately 10- and 40-fold, respectively. The elevated deoxycorticosterone levels result in the expected toxicities of secondary mineralocorticoid syndrome. Abiraterone acetate also inhibits C17,20-lyase (blunt arrow) resulting in significant declines in dehydroepiandrostenedione (DHEA), androstenedione and testosterone. Aldosterone levels fall due to suppression of the renin–angiotensin pathway by high levels of deoxycorticosterone. However, there is a four-fold increase in 11-deoxycortisol, which may be due to the increased ACTH levels driving the partial reversal of the activity of 17*α*-hydroxylase but not C17,20-lyase.

The CYP17 enzyme localises to the endoplasmic reticulum of Leydig cells in the testis, theca interna region of the ovaries, and zona fasciculata and reticularis in the adrenal glands. It is a key enzyme in the generation of androgens and oestrogens in the adrenal glands and tumour tissue, and works by the catalysis of two independently regulated steroid reactions, involving 17*α*-hydroxylase and C17, 20-lyase, in the biosynthesis pathway, as illustrated in Figure 1 (Miller *et al*, 1997). The 17*α*-hydroxylase activity converts pregnenolone to 17*α*-hydroxypregnenolone and progesterone to 17*α*-hydroxyprogesterone, whereas C17,20-lyase converts 17*α*-hydroxypregnenolone to dehydroepiandrosterone (DHEA) and 17*α*-hydroxyprogesterone to androstenedione (Miller *et al*, 1997; Auchus, 2004).

### Rationale for the safe inhibition of CYP17

In congenital CYP17 deficiencies, the production of cortisol, androgens and oestrogens is impaired, leading to the lack of sexual development (Auchus, 2004). Nonetheless, the synthesis of corticosterone, a weaker glucocorticoid, is preserved. Hence, patients do not develop symptoms of adrenal insufficiency. However, higher levels of adrenocorticotrophic hormone (ACTH) are required before a new steady state is reached, as corticosterone is a weaker glucocorticoid than cortisol. Raised levels of ACTH result in a syndrome of secondary mineralocorticoid excess characterised by fluid overload, hypertension and hypokalaemia (Figure 1). Fortuitously, this syndrome may be managed effectively with mineralocorticoid antagonists, with or without low dose glucocorticoids to suppress ACTH generation. ACTH suppression, in turn, leads to normalisation of mineralocorticoid levels, and thence, serum potassium levels and blood pressure.

### Prior experience with ketoconazole

Ketoconazole, an antifungal with weak and non-specific CYP17 inhibitory properties, has been extensively used for the ‘off-label’ treatment of advanced CRPC. The effective inhibition of the 14*α*-demethylase enzyme (that is responsible for its antifungal properties) occurs at low doses of ketoconazole, whereas much higher doses are required for the non-specific inhibition of CYP17 (De Coster *et al*, 1986) and 11*β*-hydroxylase (Loose *et al*, 1983), but this results in neurological, respiratory and hepatic toxicities which are common and severe, with up to 20% of patients discontinuing treatment.

Efficacy data from phase II trials have shown that the response rate by prostate specific antigen (PSA) working group (PSAWG) criteria (Bubley *et al*, 1999) with ketoconazole range between 40–62% with a median duration of up to 7 months (Small *et al*, 1997; Millikan *et al*, 2001; Figg *et al*, 2005; Ryan *et al*, 2007). The CALGB 9583 phase III study compared antiandrogen withdrawal alone *vs* antiandrogen withdrawal plus ketoconazole 400 mg thrice daily, resulting in response rates by PSAWG of 11% *vs* 27%, respectively, but no difference in overall survival; the latter observation was thought to be attributable, at least in part, to a high crossover rate of 82% to the ketoconazole arm (Small *et al*, 2004). It was also noted that resistance to ketoconazole can develop rapidly due to the loss of CYP17 inhibitory effect (Small *et al*, 2004). In addition, most prospective studies incorporate the use of corticosteroids; this does not seem to reduce toxicities from high-dose ketoconazole and has additionally complicated the interpretation of the endocrine and antitumoural effects.

Overall ketoconazole has antitumoural activity in prostate cancer. Although its specific role in prostate cancer therapeutics remains debatable, results from studies using it argued for the potentially important role of more selective and irreversible CYP17 inhibitors.

### Preclinical development of abiraterone acetate

**Figure 2:**
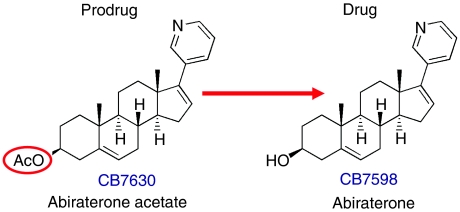
Chemical structures of abiraterone acetate and abiraterone.

The pregnenolone-derived compound, abiraterone (CB7598), was developed as part of a series of potent, inhibitory 17-(3-pyridyl) steroids at The Institute of Cancer Research, UK (Barrie *et al*, 1994; Potter *et al*, 1995; Rowlands *et al*, 1995). The features which set it apart from the other candidate small molecular inhibitors of the CYP17 enzyme included a 3-pyridyl substituent and a double-bond between positions 16 and 17 of the steroidal skeleton, as illustrated in Figure 2; these render both potency and selectivity in CYP17 inhibition to this compound with a Ki_app_ of <1 nM. The acetate prodrug, CB7630, was recommended for oral dosing due to its improved bioavailability and favourable pharmacokinetic profile.

In preclinical toxicology studies, abiraterone reduced the weights of androgen-dependent organs (prostate, seminal vesicles and testes) with minimal side effects in other organs when administered daily.

### Phase I studies of abiraterone acetate

First-in-man phase I studies reported that abiraterone acetate was safe when administered daily for 12 days to men with non-progressing prostate cancer, and resulted in >50% suppression of baseline testosterone in non-castrate patients (O’Donnell *et al*, 2004). However, this effect was transient and was negated by a compensatory rise in luteinizing hormone levels within 3 days. Hence, concomitant castration was maintained in subsequent studies to prevent this.

On the background of the need for an improved means of depleting androgens, coupled with the presence of a sound rationale for the safety of continuous inhibition of CYP17, alongside preliminary evidence for the favourable pharmacokinetic and endocrine profiles of abiraterone acetate, we performed a phase I clinical trial that has been recently reported (Attard *et al*, 2008). This involved the continuous, once-daily dosing of abiraterone acetate to castrate, chemotherapy-naïve patients with CRPC, escalating through five pre-planned dose levels of 250, 500, 750, 1000 and 2000 mg using three-patient cohorts. This was the first study to show that selective and continuous inhibition of CYP17 is safe with an associated durable suppression of serum androgens and oestrogens, as well as durable antitumour activity. This study was not designed to compare the antitumour activity of different dose-levels; as no treatment-related grade 3 or 4 toxicities were encountered and clinical responses were reported at all dose levels tested, the recommended phase II dose of 1000 mg was selected on the basis of the observation that the rise of upstream steroids reached a plateau at doses above 750 mg.

The toxicities observed in this study were predominantly due to secondary mineralocorticoid excess and were controlled with the mineralocorticoid receptor antagonist, eplerenone; spironolactone was not used as it has been previously reported to activate AR (Luthy *et al*, 1988). In keeping with the described congenital syndromes of CYP17 deficiency, no patient developed clinically significant adrenocortical insufficiency. Circulating testosterone levels in all patients were in the castrate range at baseline and rapidly became undetectable on treatment at all dose-levels. Indeed, downstream C-21 androgenic steroids were suppressed to levels below the lower limit of detection by conventional assays on treatment.

Overall, 66% of the CRPC patients treated in this study had a ⩾30% fall in PSA. Declines in PSA were frequently associated with symptomatic improvement, reduction in serum alkaline phosphatase levels, normalisation of elevated lactate dehydrogenase levels and partial responses by RECIST, with several patients reducing or discontinuing analgesic (including opiate) use. Significantly, tumour responses to abiraterone acetate were observed in castrate patients who had failed several prior lines of AR-targeting therapy (median of three prior hormonal therapies). Subsequent phase I/II studies in the UK and US have treated more than 100 patients and provide additional support to the efficacy of this agent. These preliminary efficacy data compare favourably with established second-line ADT and novel molecularly targeting agents, and mandate further evaluation of this agent in randomised studies.

### Phase II/III studies of abiraterone acetate

The results of the phase I studies have led to the phase II evaluation of abiraterone acetate in CRPC patients in the UK and US (Danila *et al*, 2008; de Bono *et al*, 2008; Reid *et al*, 2008). Specifically, the efficacy of abiraterone in the settings of both taxane-naïve and taxane-resistant CRPC have been examined, the results of which will soon be fully reported. We have hypothesised that the antitumour activity of abiraterone acetate will not be significantly affected by previous treatment with docetaxel and that abiraterone acetate could be an efficacious treatment in docetaxel-resistant disease. Indeed, preliminary analyses suggest that this is likely to be true.

A placebo-controlled randomised phase III study with the aim of securing FDA licensing approval in the post-docetaxel setting is now open to accrual; 1180 patients will be randomised 2 : 1 for abiraterone acetate plus prednisolone (or prednisone) *vs* prednisolone (or prednisone) plus placebo, with a primary endpoint of overall survival. The combination of steroids and abiraterone acetate should potentially prevent the syndrome of secondary mineralocorticoid excess and maximise efficacy.

### Roles of steroids upstream of CYP17 and abiraterone resistance

The antitumour activity reported with abiraterone acetate could be explained, at least in part, by the durable and profound suppression of serum androstenedione and DHEA, in addition to that of testosterone and oestradiol (Attard *et al*, 2008). In keeping with earlier reports regarding ketoconazole, these data from the phase I study suggest that higher baseline androstenedione and DHEA levels are present in patients who responded to abiraterone acetate. However, unlike ketoconazole, no rise in steroids downstream of CYP450c17 was observed at disease progression while on abiraterone acetate, indicating ongoing, irreversible CYP450c17 inhibition.

We hypothesised, *a priori*, that acquired resistance to abiraterone acetate could be reversed by suppressing the production of steroids upstream of CYP17 and this could be achieved by decreasing ACTH using low dose steroids replacement. Indeed, 4 out of 15 patients (26%) with two of these patients having had progressive disease earlier on the same dose of dexamethasone, were successfully salvaged using this approach (Attard *et al*, 2008) in the phase I study. Hence, it is possible that elevated levels of steroids upstream of CYP450c17 could drive the signalling of a promiscuous, possibly mutated, AR.

Other mechanisms that allow for constitutive AR signalling include mutated AR; ligandless activation of AR signalling, which could occur secondary to AR amplification or following phosphatase and tensin homologue (PTEN) loss; and/or activation of translocated *ETS* gene promoter elements by other steroid receptors, such as the oestrogen receptor (ER)-*α* (Taplin, 2007). In fact, oestradiol activate ER-*α* binding sites on the TMPRSS2 promoter and the suppression of its production could, in part, explain the antitumour activity observed with abiraterone acetate (Ellem and Risbridger, 2007). Translational studies are ongoing to test these hypotheses.

### Robust biomarkers as predictive and intermediate endpoints

To maximise benefit to patients and accelerate drug approval by regulatory authorities, the evaluation of robust biomarkers that can serve as predictive and intermediate end-points is urgently required in CRPC therapeutic studies. We describe the preliminary experience we have of the two of the most promising biomarkers that are emerging in this field; the results of their prospective verification in larger cohorts of subjects are eagerly awaited.

Fusion of the androgen-regulated gene, *TMPRSS2*, with *ERG* occurs in up to 60% of prostate cancers and is likely to account for the majority of *ETS* oncogene rearrangements in prostate cancer (Attard *et al*, 2008). We therefore hypothesised that the presence of a *TMPRSS2–ERG* fusion gene could indicate dependance on AR signalling and consequently define a tumour sub-group that is responsive to abiraterone acetate. In preliminary studies, PSA decline rate appears to be higher in patients with an *ERG* rearrangement in archival trans-rectal biopsy of prostate (TRBP) samples (five out of six patients in the published phase I study with an *ERG* gene rearrangement had a decline in PSA ⩾50%). However, due to *ETS* gene rearrangement heterogeneity within a single prostate, the use of single TRBP cores may miss areas with *ERG* rearrangements and fusion of other *ETS* genes with androgen-regulated partners can also occur.

We have also recently shown that the presence of ⩾5 circulating tumour cells at baseline is associated with a poor outcome and that a fall in CTC count to <5 following treatment is associated with an improved prognosis (Olmos *et al*, 2009). Significantly, five patients in the phase I study had baseline CTC counts ⩾5 and three of these patients had their CTC counts reduced to <5 after 12 weeks of treatment. The aforementioned phase III study is therefore incorporating the prospective evaluation of whether changes in CTC counts post treatment can serve as a robust intermediate endpoint for overall survival to accelerate drug development for CRPC.

## Other agents directly targeting the AR-signalling pathway

In addition to abiraterone and ketoconazole, there are other CYP17 inhibitors that are at various stages of development. VN/124-1, currently in preclinical development and recently licensed to Tokai Pharmaceuticals Inc. (Boston, NY, USA), is a 17-benzoimidazole that has direct inhibitory properties against CYP17 and AR (Handratta *et al*, 2005). It has potent antitumour effects *in vitro* with the ability to effectively inhibit proliferation of a bicalutamide-resistant prostate cancer cell lines, which had increased AR expression. Interestingly, the combination of VN/124-1 with either everolimus or gefitinib *in vitro* was also superior to that achieved with the addition of bicalutamide to either agent, and the VN/124-1 combination effects were synergistic compared with either agent alone (Schayowitz *et al*, 2008). It is expected that this agent will be evaluated in the clinical setting soon.

The novel small molecule AR antagonist, MDV3100, was rationally designed for its ability to overcome resistance to conventional bicalutamide in the setting of increased AR expression (Sawyers *et al*, 2007). Unlike bicalutamide, MDV3100 inhibits AR function by blocking nuclear translocation and has no agonist activity when AR is overexpressed. A first-in-man, multi-center phase I/II dose-escalation study has been reported with over 50 patients having enrolled when last reported (Scher *et al*, 2008). In all, MDV3100 has been well tolerated to date with no significant adverse events. Significantly, it has resulted in PSA reductions in a high proportion of evaluable patients and appears to be a promising candidate for the treatment of progressive CRPC. This trial is currently ongoing.

To summarise, these agents work directly to reduce or inhibit AR-mediated signalling by ligand depletion and/or receptor antagonism. Overall, these data provide further mechanistic and clinical evidence of the importance and relevance of AR-mediated signalling pathway in CRPC therapeutics.

## Future directions

The promising efficacy of abiraterone acetate stemming from these early clinical trials in CRPC setting attests to our renewed recognition of the importance of the AR-signalling pathway in CRPC biology. It is sobering to realise that up to 70% of patients who had disease progression on previous lines of hormonal therapies, are still hormone dependant. Indeed, the recent revision of misnomers, such as ‘androgen independence’ and ‘hormone resistance’, by the Prostate Cancer Clinical Trials Working Group is a reflection of this. The successful reversal of resistance to abiraterone acetate using low-dose dexamethasone in some patients also points to the likely importance of steroids upstream of CYP17, and indicate that AR, although likely mutated and promiscuous, remains key in this process.

Although the numbers of subjects and biological specimens involved are small, the evidence from the translational studies involving *ETS* gene fusion products and CTCs provide further, albeit preliminary, molecular biological corroboration of the importance of AR-mediated signalling mechanisms. These novel molecular platforms may have additional utility in improving our ability to select patients more likely to benefit from abiraterone acetate treatment.

There are numerous questions emerging from the use of this compound. For instance, how are the intratumoural androgenic steroid levels affected by abiraterone acetate? What are the mechanisms of resistance to it? In what clinical settings ought the drug be used? With which agents, cytotoxic and novel, should abiraterone acetate be used? What about the role of abiraterone acetate in other hormonally driven malignancies?

Overall, these data support the development of further novel inhibitors of AR signalling. However, sceptics will trumpet the acknowledgement that we have been in similar positions before with other ADT modalities. We argue that these new clinical and translational seeds are being sowed in research soil more fertile than ever before, enriched by an improved understanding of the pathways of treatment resistance and the availability of new technologies in the evaluation of tumour biology. Hence, not only do we have more effective compounds, there are unparalleled opportunities for these to be combined with emerging novel biological agents, supported by the presence of new molecular and imaging methodologies.

## References

[bib1] Attard G, Reid AH, Yap TA, Raynaud F, Dowsett M, Settatree S, Barrett M, Parker C, Martins V, Folkerd E, Clark J, Cooper CS, Kaye SB, Dearnaley D, Lee G, de Bono JS (2008) Phase I clinical trial of a selective inhibitor of CYP17, abiraterone acetate, confirms that castration-resistant prostate cancer commonly remains hormone driven. J Clin Oncol 26: 4563–4571 18645193 10.1200/JCO.2007.15.9749

[bib2] Auchus RJ (2004) Overview of dehydroepiandrosterone biosynthesis. Semin Reprod Med 22: 281–288 15635496 10.1055/s-2004-861545

[bib3] Barrie SE, Potter GA, Goddard PM, Haynes BP, Dowsett M, Jarman M (1994) Pharmacology of novel steroidal inhibitors of cytochrome P450(17) alpha (17 alpha-hydroxylase/C17-20 lyase). J Steroid Biochem Mol Biol 50: 267–273 7918112 10.1016/0960-0760(94)90131-7

[bib4] Bubley GJ, Carducci M, Dahut W, Dawson N, Daliani D, Eisenberger M, Figg WD, Freidlin B, Halabi S, Hudes G, Hussain M, Kaplan R, Myers C, Oh W, Petrylak DP, Reed E, Roth B, Sartor O, Scher H, Simons J, Sinibaldi V, Small EJ, Smith MR, Trump DL, Wilding G et al (1999) Eligibility and response guidelines for phase II clinical trials in androgen-independent prostate cancer: recommendations from the Prostate-Specific Antigen Working Group. J Clin Oncol 17: 3461–3467 10550143 10.1200/JCO.1999.17.11.3461

[bib5] Chen CD, Welsbie DS, Tran C, Baek SH, Chen R, Vessella R, Rosenfeld MG, Sawyers CL (2004) Molecular determinants of resistance to antiandrogen therapy. Nat Med 10: 33–39 14702632 10.1038/nm972

[bib6] Danila DC, Rathkopf DE, Morris MJ, Slovin SF, Schwartz LH, Farmer K, Anand A, Haqq C, Fleisher M, Scher HI (2008) Abiraterone acetate and prednisone in patients (Pts) with progressive metastatic castration resistant prostate cancer (CRPC) after failure of docetaxel-based chemotherapy. J Clin Oncol (Meet Abstr) 26: 5019

[bib7] de Bono JS, Attard G, Reid AH, Parker C, Dowsett M, Mollife R, Yap TA, Molina A, Lee G, Dearnaley D (2008) Anti-tumor activity of abiraterone acetate (AA), a CYP17 inhibitor of androgen synthesis, in chemotherapy naive and docetaxel pre-treated castration resistant prostate cancer (CRPC). J Clin Oncol (Meet Abstr) 26: 5005

[bib8] De Coster R, Caers I, Coene MC, Amery W, Beerens D, Haelterman C (1986) Effects of high dose ketoconazole therapy on the main plasma testicular and adrenal steroids in previously untreated prostatic cancer patients. Clin Endocrinol (Oxf) 24: 657–664 2947757 10.1111/j.1365-2265.1986.tb01662.x

[bib9] Ellem SJ, Risbridger GP (2007) Treating prostate cancer: a rationale for targeting local oestrogens. Nat Rev Cancer 7: 621–627 17611544 10.1038/nrc2174

[bib10] Ferlay J, Autier P, Boniol M, Heanue M, Colombet M, Boyle P (2007) Estimates of the cancer incidence and mortality in Europe in 2006. Ann Oncol 18: 581–592 17287242 10.1093/annonc/mdl498

[bib11] Figg WD, Liu Y, Arlen P, Gulley J, Steinberg SM, Liewehr DJ, Cox MC, Zhai S, Cremers S, Parr A, Yang X, Chen CC, Jones E, Dahut WL (2005) A randomized, phase II trial of ketoconazole plus alendronate versus ketoconazole alone in patients with androgen independent prostate cancer and bone metastases. J Urol 173: 790–796 15711271 10.1097/01.ju.0000147013.09157.8e

[bib12] Handratta VD, Vasaitis TS, Njar VC, Gediya LK, Kataria R, Chopra P, Newman Jr D, Farquhar R, Guo Z, Qiu Y, Brodie AM (2005) Novel C-17-heteroaryl steroidal CYP17 inhibitors/antiandrogens: synthesis, *in vitro* biological activity, pharmacokinetics, and antitumor activity in the LAPC4 human prostate cancer xenograft model. J Med Chem 48: 2972–2984 15828836 10.1021/jm040202w

[bib13] Loose DS, Kan PB, Hirst MA, Marcus RA, Feldman D (1983) Ketoconazole blocks adrenal steroidogenesis by inhibiting cytochrome P450-dependent enzymes. J Clin Invest 71: 1495–1499 6304148 10.1172/JCI110903 PMC437014

[bib14] Luthy IA, Begin DJ, Labrie F (1988) Androgenic activity of synthetic progestins and spironolactone in androgen-sensitive mouse mammary carcinoma (Shionogi) cells in culture. J Steroid Biochem 31: 845–852 2462135 10.1016/0022-4731(88)90295-6

[bib15] Miller WL, Auchus RJ, Geller DH (1997) The regulation of 17,20 lyase activity. Steroids 62: 133–142 9029728 10.1016/s0039-128x(96)00172-9

[bib16] Millikan R, Baez L, Banerjee T, Wade J, Edwards K, Winn R, Smith TL, Logothetis C (2001) Randomized phase 2 trial of ketoconazole and ketoconazole/doxorubicin in androgen independent prostate cancer. Urol Oncol 6: 111–115 11344001 10.1016/s1078-1439(00)00123-x

[bib17] O’Donnell A, Judson I, Dowsett M, Raynaud F, Dearnaley D, Mason M, Harland S, Robbins A, Halbert G, Nutley B, Jarman M (2004) Hormonal impact of the 17alpha-hydroxylase/C(17,20)-lyase inhibitor abiraterone acetate (CB7630) in patients with prostate cancer. Br J Cancer 90: 2317–2325 15150570 10.1038/sj.bjc.6601879 PMC2409523

[bib18] Olmos D, Arkenau HT, Ang JE, Ledaki I, Attard G, Carden CP, Reid AH, A’Hern R, Fong PC, Oomen NB, Molife R, Dearnaley D, Parker C, Terstappen LW, de Bono JS (2009) Circulating tumour cell (CTC) counts as intermediate end points in castration-resistant prostate cancer (CRPC): a single-centre experience. Ann Oncol 20: 27–33 10.1093/annonc/mdn544 18695026

[bib19] Pienta KJ, Bradley D (2006) Mechanisms underlying the development of androgen-independent prostate cancer. Clin Cancer Res 12: 1665–1671 16551847 10.1158/1078-0432.CCR-06-0067

[bib20] Potter GA, Barrie SE, Jarman M, Rowlands MG (1995) Novel steroidal inhibitors of human cytochrome P45017 alpha (17 alpha-hydroxylase-C17,20-lyase): potential agents for the treatment of prostatic cancer. J Med Chem 38: 2463–2471 7608911 10.1021/jm00013a022

[bib21] Raghavan D, Klein EA (2008) Prostate cancer: moving forward by reinventing the wheel...but this time it is round. J Clin Oncol 26: 4535–4536 18626003 10.1200/JCO.2008.18.3145

[bib22] Reid AH, Attard G, Molife R, Olmos D, Babu ON, Thompson E, Parker C, Dearnaley D, Lee G, De-Bono JS (2008) Selective CYP17 inhibition with abiraterone acetate (AA) results in a high response rate (RR) in castration-resistant prostate cancer (CRPC) confirming the continued importance of targeting androgen receptor signaling. Genitourinary Cancer Symposium San Francisco, CA, USA. Abstract: 50

[bib23] Rowlands MG, Barrie SE, Chan F, Houghton J, Jarman M, McCague R, Potter GA (1995) Esters of 3-pyridylacetic acid that combine potent inhibition of 17 alpha-hydroxylase/C17,20-lyase (cytochrome P45017 alpha) with resistance to esterase hydrolysis. J Med Chem 38: 4191–4197 7473546 10.1021/jm00021a008

[bib24] Ryan CJ, Weinberg V, Rosenberg J, Fong L, Lin A, Kim J, Small EJ (2007) Phase II study of ketoconazole plus granulocyte-macrophage colony-stimulating factor for prostate cancer: effect of extent of disease on outcome. J Urol 178: 2372–2376; discussion 2377 17936834 10.1016/j.juro.2007.08.011

[bib25] Sawyers CL, Tran C, Wongvipat J, Ouk S, Yoo D, Protter AA, Hung DT, Jung ME (2007) Characterization of a new anti-androgen MDV-3100 effective in preclinical models of hormone refractory prostate cancer. Prostate Cancer Symposium Orlando, FL, USA. Abstract: 48

[bib26] Schayowitz A, Sabnis G, Njar VC, Brodie AM (2008) Synergistic effect of a novel antiandrogen, VN/124-1, and signal transduction inhibitors in prostate cancer progression to hormone independence *in vitro*. Mol Cancer Ther 7: 121–132 18202015 10.1158/1535-7163.MCT-07-0581

[bib27] Scher HI, Beer TM, Higano CS, Danila DC, Montgomery B, Shelkey J, Hirmand M, Hung D, Sawyers C (2008) Phase I/II study of MDV3100 in patients (pts) with progressive castration-resistant prostate cancer (CRPC). J Clin Oncol (Meeting Abstracts) 26: 5006

[bib28] Small EJ, Baron A, Bok R (1997) Simultaneous antiandrogen withdrawal and treatment with ketoconazole and hydrocortisone in patients with advanced prostate carcinoma. Cancer 80: 1755–1759 9351544 10.1002/(sici)1097-0142(19971101)80:9<1755::aid-cncr9>3.0.co;2-d

[bib29] Small EJ, Halabi S, Dawson NA, Stadler WM, Rini BI, Picus J, Gable P, Torti FM, Kaplan E, Vogelzang NJ (2004) Antiandrogen withdrawal alone or in combination with ketoconazole in androgen-independent prostate cancer patients: a phase III trial (CALGB 9583). J Clin Oncol 22: 1025–1033 15020604 10.1200/JCO.2004.06.037

[bib30] Stanbrough M, Bubley GJ, Ross K, Golub TR, Rubin MA, Penning TM, Febbo PG, Balk SP (2006) Increased expression of genes converting adrenal androgens to testosterone in androgen-independent prostate cancer. Cancer Res 66: 2815–2825 16510604 10.1158/0008-5472.CAN-05-4000

[bib31] Taplin ME (2007) Drug insight: role of the androgen receptor in the development and progression of prostate cancer. Nat Clin Pract Oncol 4: 236–244 17392714 10.1038/ncponc0765

[bib32] Titus MA, Schell MJ, Lih FB, Tomer KB, Mohler JL (2005) Testosterone and dihydrotestosterone tissue levels in recurrent prostate cancer. Clin Cancer Res 11: 4653–4657 16000557 10.1158/1078-0432.CCR-05-0525

